# Transforaminal Lumbar Interbody Fusion Using a Modified Distractor Handle: A Midterm Clinicoradiological Follow-Up Study 

**DOI:** 10.1155/2013/926094

**Published:** 2013-09-09

**Authors:** Abuduaini Rewuti, Zixian Chen, Zhenzhou Feng, Yuanwu Cao, Xiaoxing Jiang, Chun Jiang

**Affiliations:** Department of Orthopedics, Zhongshan Hospital, Fudan University, 180 Fenglin Road, Shanghai 20032, China

## Abstract

In current TLIF practice, the choice of the cage size is empirical and primarily depends on the case volume and experience of the surgeon. We used a self-made modified distractor handle in TLIF procedure with the goal of standardizing the intervertebral space tension and determining the proper cage size.

## 1. Introduction

Lumbar interbody fusion (LIF) is the gold standard treatment modality for lumbar degenerative disease (LDD) [1]. LIF aims to create a reliable spinal arthrodesis that can bear lumbar load, maintain the intervertebral space and foraminal dimension, and restore normal sagittal plane alignment [2]. Recent years have been marked by major breakthroughs in terms of the operative technique, instrumentation, and bone graft materials used for LIF. Whereas anterior, posterior, and lateral LIF (ALIF, PLIF, and LLIF, resp.) were once frequently performed, concerns have been raised regarding the procedural safety of these techniques, due to their aggressive invasiveness and high risk of iatrogenic injury, such as dural tearing, epidural bleeding, and neural damage [3, 4].

Transforaminal LIF (TLIF), an alternative technique to ALIF and PLIF, was first reported by Blume [5] in the early 1980s and was advocated by Harms and Rolinger [6] in 1997. In TLIF, only the ipsilateral foramen is exposed by using pedicle screws before cage insertion. This approach minimizes damage to the perilumbar soft tissues [7]. Moreover, the thecal sac is limitedly retracted, which reduces the risk of neurological injury [7]. Previous clinical studies comparing TLIF and PLIF demonstrated that TLIF achieved clinical outcomes and circumferential fusion comparable to PLIF [8]. Additional biomechanical studies confirmed that TLIF offered favorable postfusion mechanical stability and segmental flexibility similar to those of PLIF [9]. Refinements in interbody fusion devices and rod-screw fixation systems have broadened the indications for TLIF, which now include symptomatic spondylolisthesis [10], degenerative scoliosis [11], spinal stenosis [12], and recurrent lumbar disc herniation [13].

TLIF is subject to some technical limitations. The entire removal of facet joints may cause recurrent low back pain and spinal deformity. Implantation of two cages is normally required to restore segmental alignment and achieve reliable fusion, although the use of a single cage has been reported [14]. In most cases, bone grafting is required to ensure 360° fusion. More importantly, in current TLIF practice, the choice of the cage size is empirical and primarily depends on the case volume and experience of the surgeon. However, use of an oversized cage may result in increased tension of the intervertebral space and consequent endplate collapse [15]. An undersized cage cannot maintain interbody fusion, leading to pseudarthrosis formation and fusion failure [16, 17]. Thus, accurate measurement of the intervertebral disc space height (IVDSH) is paramount for the appropriate choice of cage size and the success of TLIF.

In our spinal surgery unit, we have been replacing the conventional T-handle and spreader curette with a modified, scaled (0–6 N) distractor handle, with the goal of standardizing the intervertebral space tension and determining the proper cage size. The objective of this retrospective study was to examine the clinical and radiological results of TLIF performed with this modified distractor handle for the treatment of LDD in a midterm follow-up study.

## 2. Patients and Methods

### 2.1. Patients

**Figure 1:**
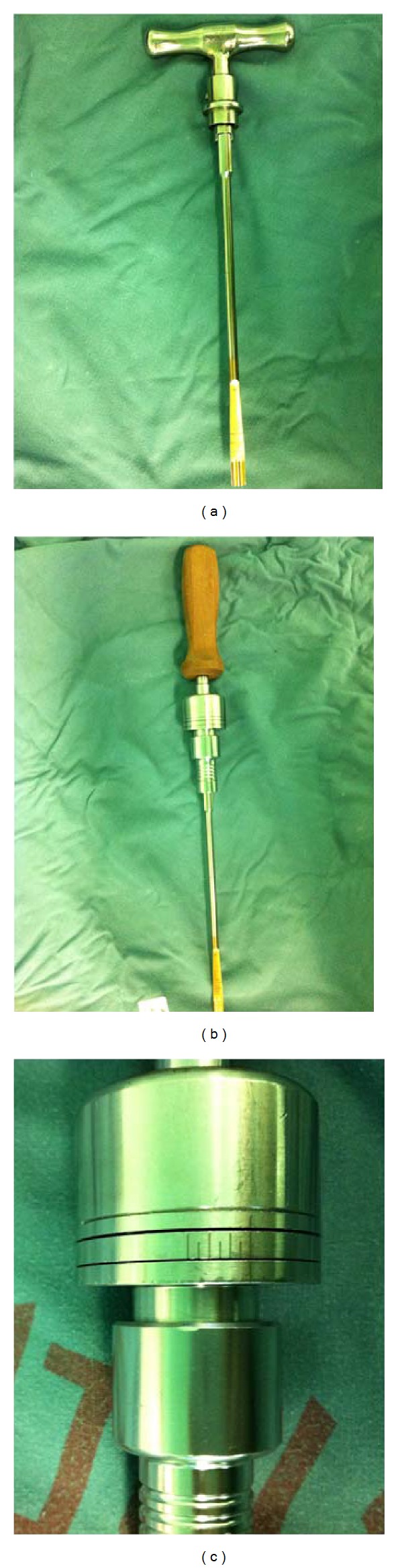
Conventional T-handle (a) and the modified, scaled distractor handle (b) used for the selection of cage size with a tension scale of 0–6 N from right to left, 1 N per scale (c).

The study protocol was approved by the Institutional Review Board at Zhongshan Hospital, Fudan University, Shanghai, China. This retrospective study included 45 patients (30 men; 15 women mean age, 53 years; range, 40–73 years) with radiographically documented LDD who were consecutively hospitalized and scheduled for elective unilateral, instrumented TLIF with the modified distractor handle (Figures 1(a) and 1(b)) at the authors' spinal surgery unit between March 2009 and March 2010. Chief complaints, including low back pain, unilateral radiculopathy, and intermittent claudication, did not markedly improve with conservative management. The surgical conditions were as follows: degenerative disc disease with a specific discogenic pain pattern; recurrent lumbar disc herniation with radiculopathy, regardless of the presence or absence of low back pain; lumbar stenosis with instability or complex lumbar stenosis; or grade 1 or 2 spondylolisthesis. No patient had undergone previous surgical intervention, such as posterior decompression, and no patient was known to have severe osteoporosis. 

### 2.2. Preoperative Assessment

Routine perioperative medical evaluation was performed in accordance with the patient's physical status, which was classified by the system of the American Society of Anesthesiologists. In no patient was TLIF contraindicated by any medical or surgical condition. LDD and concomitant lumbar conditions were confirmed by anteroposterior and lateral (flexion and extension) lumbar spine radiography, computed tomography (CT), and magnetic resonance imaging. Complicating lumbar conditions were identified in all patients, which included the following: concomitant spinal stenosis with lumbar instability in 25 patients, involving a single segment (*n* = 19) or two segments (*n* = 6); spondylolisthesis involving a single segment in 20 patients, including grade 1 (*n* = 11) or grade 2 (*n* = 9) spondylolisthesis. All patients gave their written informed consent prior to participation in this study. All operations were performed by an assigned surgical team led by the corresponding author.

### 2.3. Operative Technique

Unilateral instrumented TLIF was performed *via* open access as previously described [18]. Briefly, patients were intubated under general anesthesia and positioned supine with the hip joints fully extended to maintain the lumbar convex curvature. Antimicrobial prophylaxis was given 30 minutes prior to making the skin incision. C-arm fluoroscopy was used to determine the surface projection of the involved pedicles. A paramedian skin incision was made, paralleling the involved pedicles on the symptomatic side, at 2-3 cm from the spinous process and overlying the facet joints. The skin, subcutaneous adipose tissue, and lumbodorsal fascia were successively incised. The erector spinae muscle fibers were split to expose the facet joints, lamina isthmus, and transverse process. The pedicle screws were instrumented, and the ipsilateral facets and part of the upper level lamina were excised. The *ligamentum flavum* was dissected to expose the superior and inferior nerve roots and the dura mater. The nerve roots were well preserved, and the annulus fibrosus was incised. A box cutter was used to open the entry into the disc space for a thorough discectomy and endplate curettage. The endplate cartilage was removed completely, whereas the subendplate bone was well preserved.

**Figure 2:**
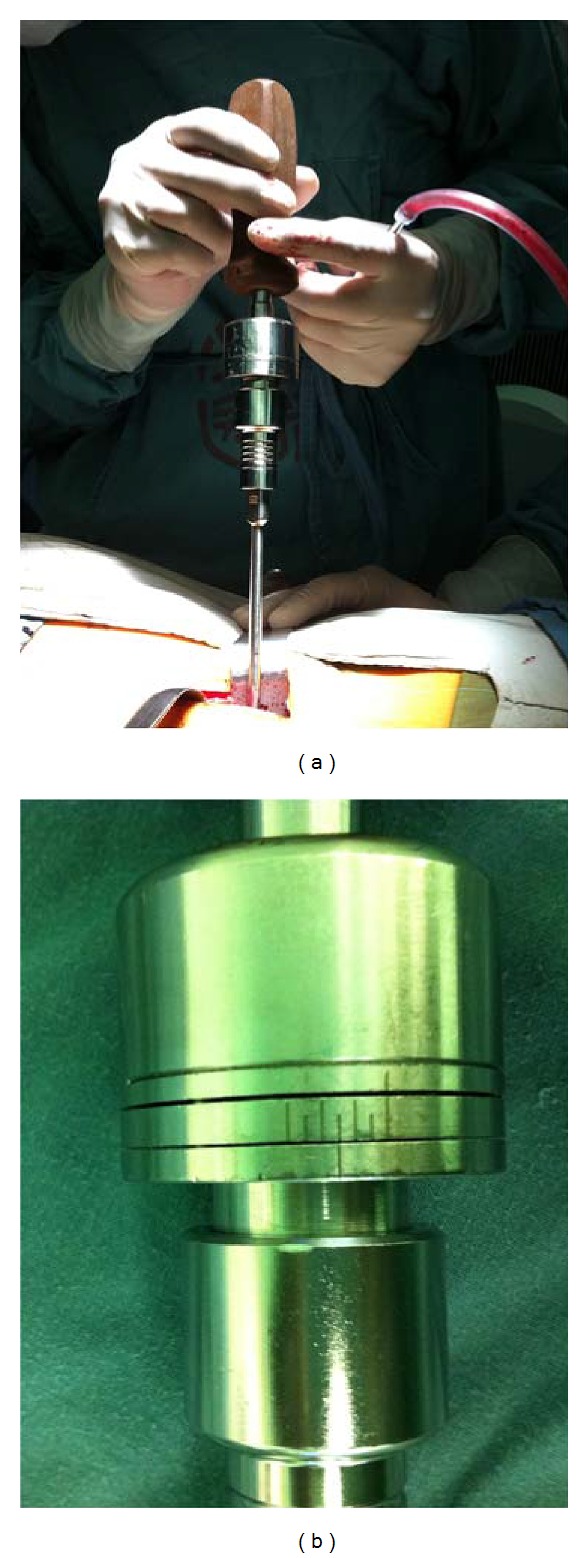
The self-made modified distractor handle was used to measure the intervertebral space height (a). The handle was spanned clockwise to increase the distraction tension, and cage size was determined with the distractor handle maintained at 2–4 N (b).

A preliminary study was done to measure the intervertebral space distraction tension in this study. Briefly, following endplate dissection the intervertebral space was distracted using the T-handle and an appropriate-sized cage was placed. Then the self-made modified, scaled (0–6 N) distractor handle was used to measure the intervertebral space tension, 1 N per scale. The distraction tension turned out to be 2–4 N (median, 3 N) in the great majority of patients. In subsequent experiment, the self-made modified distractor handle was used to measure the IVDSH for the selection of proper cage size, with the distractor handle tension maintained at 2 to 4 N (median, 3 N), as determined by the preliminary results (Figures 1(b) and 1(c)). A self-made modified distractor handle was used to measure the IVDSH for the selection of proper cage size (Figure 2(a)). The distractor handle was connected to a #6 disc spanner (the smallest-sized spanner). If the intervertebral space tension was 1-2 N, then a larger-sized spanner was used to repeat the measurement. If the tension was 3-4 N, then a cage with the same size as that of the spanner was used (Figure 2(b)). If the tension was above 4 N, then the measurement was repeated with a smaller-sized spanner. 

An appropriately sized polyether ether ketone (PEEK) cage (Stryker Corporation, Kalamazoo, MI) filled with excised local bones was inserted into the disc space to ensure a solid interbody fusion. Intraoperative lateral lumbar fluoroscopy was performed to confirm the cage position. The interbody graft was posteriorly compressed, and the pedicle screws were tightened unilaterally to restore lordosis. A drainage tube was placed prior to incision closure and removed at 24 to 48 hours after the operation. Patients were instructed to wear orthoses and start off-bed activities at 3 days after TLIF. Routine bed lumbodorsal muscle exercises were recommended.

### 2.4. Clinical and Radiologic Assessment

Patients were followed up at outpatient clinics at 3, 6, 12, and 24 months after TLIF. Clinical outcomes were assessed in a self-reported manner with the Oswestry disability index (ODI) [19] and a 10-point visual analog scale (VAS) for low back pain (0–10, from “no pain” to “extreme pain”) [20]. Medical charts were reviewed to identify any adverse events or complications, as previously reported [21]. Follow-up anteroposterior and lateral radiographs were taken 1 week after TLIF and during the aforementioned outpatient visits. Additional dynamic lateral flexion-extension radiographs were ordered at 6, 12, and 24 months after TLIF. 

Bony fusion was defined as the presence of bone trabeculae across the interfaces between the cage and the endplates without any lucencies and the formation of a bony union between the superior and inferior endplates on the lateral plain radiographs [14]. Three-dimensional CT was used to verify bony fusion at 12 and 24 months after TLIF. An independent spinal surgeon measured the IVDSH, defined as the average of the anterior and posterior heights [14]. 

### 2.5. Statistical Analysis

All data were processed with the SPSS software package, version 19.0 (SPSS Inc., Chicago, IL). Data are reported as the mean ± standard deviation (SD) for ODI, VAS, and IVDSH results. Differences in the ODI, VAS, and IVDSH results between the baseline and follow-up times were compared by one-sample repeated-measures Student's *t*-tests. A *P*  value  <0.05 was considered statistically significant.

## 3. Results

### 3.1. Clinical Results

The clinical results are shown in Table 1. The fused levels included L3-L4 (*n* = 7), L4-L5 (*n* = 23), and L5-S1 (*n* = 15). IVDSH was measured in all patients with the tension of the distractor handle maintained at 2 to 4 N (median, 3 N). All cages were successfully implanted in a single attempt. No dural or nerve tears occurred during the process of implantation. The mean operative time was 92.3 ± 34.5 minutes (range, 80–145 minutes). The mean volume of intraoperative blood loss was 120.4 ± 40.4 mL (95–160 mL). 

Postoperative recovery was uneventful for all patients. No patient complained of any postoperative neurological impairment. No delayed bleeding or surgical site infection was observed postoperatively. Wound drainage tubes were removed, and patients started off-bed activities while wearing orthoses as scheduled. The mean time of postoperative hospitalization was 7.3 days (5–12 days). The mean follow-up duration was 17.6 months (range, 12–24 months). No patient was lost to follow-up.

### 3.2. ODI and Low Back Pain VAS

**Figure 3:**
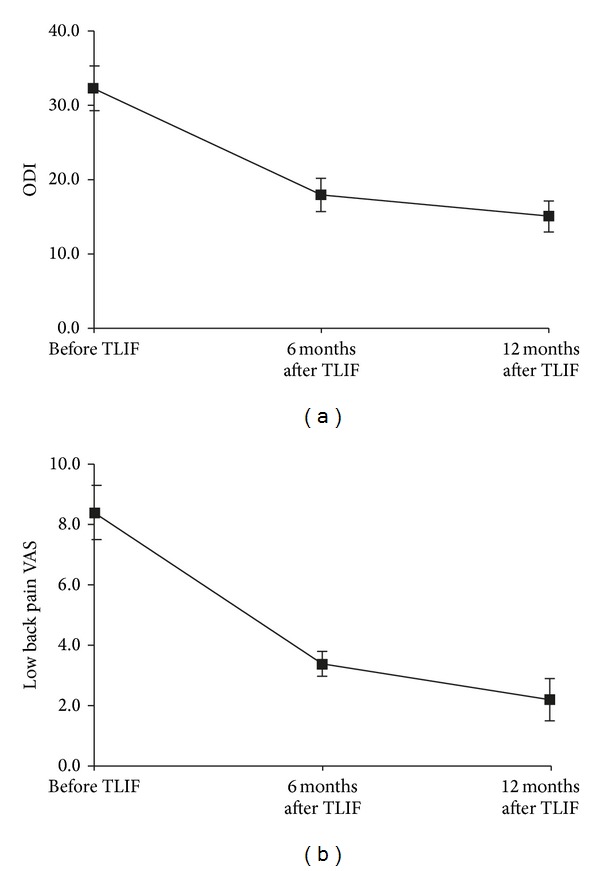
Improvements in ODI (a) and low back pain VAS (b) at 6 and 12 months after TLIF as compared to the baseline (Pre-TLIF). ODI, Oswestry disability index; VAS, visual analog scale.

All patients completed the self-reported questionnaires as instructed. The ODI score decreased from 32.3 ± 3.0 at baseline to 18.0 ± 2.2 at 6 months and 15.1 ± 2.1 at 12 months after-TLIF (both *P* < 0.01 versus baseline, Figure 3(a)). The low back pain VAS score improved from 8.4 ± 0.9 at baseline to 3.4 ± 0.4 at 6 months and 2.2 ± 0.7 at 12 months after-TLIF (both *P* < 0.01 versus baseline; Figure 3(b)). Improvements in the ODI and VAS scores continued between 6 and 12 months after-TLIF (both *P* < 0.01). No patient complained of any impairment of his or her daily activities throughout the follow-up period.

### 3.3. Radiological Results

**Figure 4:**
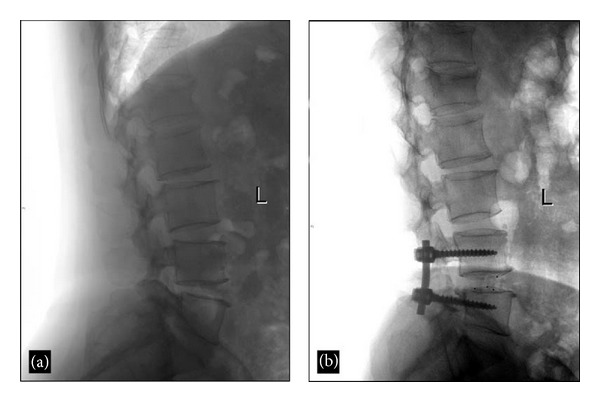
Lateral radiographs of the lumbar spine in a 55-year-old male with degenerative spondylolisthesis. (a) Intervertebral space at the L4-L5 level was narrowed, as shown on preoperative radiography. (b) The intervertebral space was restored after TLIF.

**Figure 5:**
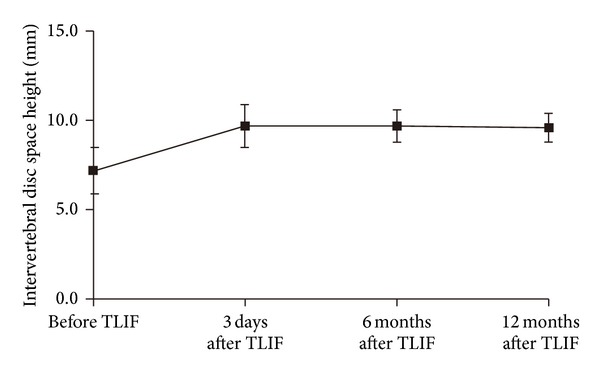
Improvements in IVDSH at 3 days, and at 6 and 12 months after TLIF as compared to the baseline (IVDSH, intervertebral disc space height).

**Figure 6:**
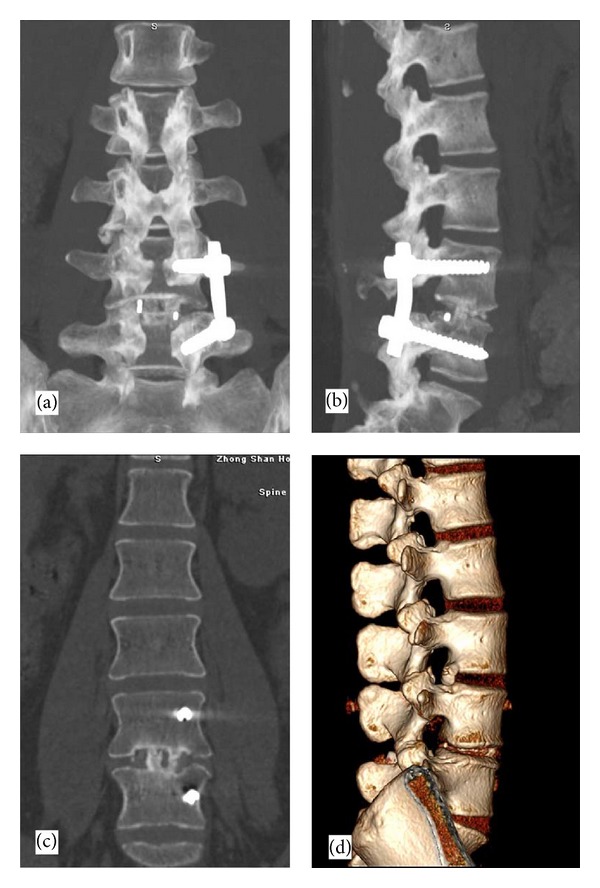
Follow-up three-dimensional CT scan revealing bony fusion at 12 months after TLIF. (a) Anteroposterior view; (b) lateral view; (c) coronal view; (d) lateral view with three-dimensional holographic reconstruction.

No fixation device failure, cage migration, or endplate collapse was observed on the follow-up radiographs. The mean IVDSH increased from 7.2 ± 1.3 mm at baseline (Figures 4(a) and 4(b)) to 9.7 ± 1.2, 9.7 ± 0.9, and 9.6 ± 0.8 mm immediately, 6 months, and 12 months after-TLIF, respectively (all *P* < 0.05 versus baseline, Figure 5). The improvement in IVDSH was well-maintained between 6 and 12 months after-TLIF (*P* > 0.01). Radiological bony fusion was achieved in 43 patients (91.6%) at 12 months and in all 45 patients (100.0%) at 24 months after-TLIF (Figures 6(a)–6(d)). 

## 4. Discussion

Currently, TLIF is well accepted as a treatment modality for various spinal disorders, including disc degenerative disease, spondylolisthesis [10], and lumbar stenosis with instability [22]. This surgical approach shows a comparable biomechanical outcome but less invasiveness as compared to PLIF [23]. Minimally invasive TLIF has become popular due to its recovery benefit, especially for obese patients. However, fusion failure still occurs in some patients who undergo TLIF and is mainly derived from intervertebral space collapse or cage migration. Collapse of the intervertebral space will destroy the restored spinal curvature, and migration of the posterior cage may result in serious neurological impairment if the nerve roots or dura mater is involved. The risk factors contributing to intervertebral space collapse or cage migration include cage size and shape, disc height, spinal curvature, number of fused segments, and endplate shape [16, 24]. Therefore, the choice of a properly sized cage is critical for fusion success in TLIF. We attempted to standardize the choice of cage size by using a modified distractor handle, obtaining favorable midterm clinical and radiological results in a retrospective patient cohort. To the best of our knowledge, this study is the first report regarding the standardization of cage size choice by using a distractor handle.

No consensus has been reached regarding the measurement of IVDSH for determining cage size. The IVDSH is usually estimated based on the average of the anterior and posterior IVDSHs, or of the superior- and inferior-segment IVDSHs, from the preoperative lateral lumbar radiographs. In conventional TLIF, a nonscaled T-handle is used to twist the disc spanner for measuring the IVDSH. The determination of the intervertebral space tension depends on the experience of the surgeon. Therefore, the choice of cage size is more empirical than standardized, particularly for inexperienced spinal surgeons. In contrast, the use of the modified distractor handle can maintain a reasonable intervertebral space stress and preserve the bony endplate, thereby minimizing endplate bleeding and the risk of interbody subsidence. Our patients lost a mean blood volume of only 120 mL intraoperatively, substantially less than the ~300 mL of blood reported in previous studies [14]. The use of a standardized cage size avoids the repeated matching of the cage and intervertebral space, dramatically shortening the operative time. Unilateral instrumented TLIF normally lasts more than 2 hours [14], whereas TLIF using the distractor handle was completed within 1.5 hours. The shortened operative time may accelerate postoperative recovery and benefit patients physically, psychologically, and medicofinancially. The ODI and low back pain VAS instruments are well-validated measures for evaluating the clinical efficacy of TLIF by comparing baseline and postoperative symptoms [19, 20]. Our patients showed significant improvements in ODI and VAS scores compared to baselines, which were maintained for up to 12 months, similar to previous reports.

Titanium cages have a much higher elasticity modulus than the vertebral body. Use of an oversized cage will increase the intervertebral stress and cause cage subsidence into the endplate, especially in osteoporotic patients [25]. Protection of the underlying bony endplate by an appropriately sized cage plays an essential role in the process of interbody bony fusion. The collapse rate of the intervertebral space was reported to be 16.2% with a titanium cage and 8.7% with a carbon fiber-reinforced polymer (CFEP) cage [26]. PEEK cages are more frequently employed in current practice, due to their extraordinary biomechanical advantages [27]. In the present study, we obtained a zero rate of cage subsidence when the PEEK cage was used. The IVDSH was well restored and maintained in the midterm follow-up period. It was superior to the IVDSH obtained with the titanium cage [26] and similar to that obtained with the CFEP cage [26]. These results may be attributed to the proper postdistraction tension of the intervertebral space and appropriate selection of cage size.

Cage migration is the primary safety concern in TLIF, due to the serious neurological consequences if the cage protrudes posteriorly into the spinal canal [24]. The rate of cage migration varies among centers and reports, ranging from 1.2% [16] to 23% [28]. Risk factors include cage shape (rectangular versus kidney shaped), cage size (small versus large), cage material (bioabsorbable versus carbon fiber) [29], number of fused segment (double versus single), endplate type (linear versus concave-concave), and instrumentation (unilateral versus bilateral) [16]. Cage migration also leads to pseudarthrosis formation and fusion failure. Proper cage size choice, in addition to appropriate endplate preparation, will conform the implant to the endplates and maximize the contact between the graft and endplates under a reasonable fusion stress, permitting a successful 360° fusion. Our results showed that no cage migration occurred, and bony fusion was observed in all patients within the 12-month follow-up period. This fusion success rate is superior to that of the titanium cage [26] and similar to that of the carbon fiber cage [27].

This study has some limitations. First, as a retrospective, single-treatment-arm study, no comparative data were present, although we did compare our postoperative results with the baseline data and against findings in the literature. Second, this was a nonblinded study; therefore, the findings are subject to the investigator's bias. However, the clinical results were assessed in a patient self-reported manner, and the radiological results were evaluated by an independent spinal surgeon who was not involved in the study. Finally, a midterm follow-up period was used, which is possibly not sufficiently long to evaluate the surgical results for such a chronic condition as LDD. A long-term clinicoradiological follow-up study is ongoing at our institute.

In conclusion, the use of a distractor handle in TLIF can decrease blood loss and operative time while showing favorable clinical improvements. The major benefit of this modification lies in the standardized and unified selection of cage size in TLIF. The proper choice of cage size using the modified distractor handle resulted in a zero rate of cage subsidence or migration and successful bony fusion in the midterm. This technique may be an effective and safe adjuvant method to TLIF for the treatment of LDD. Prospective, controlled, comparative studies are needed to evaluate the effectiveness and safety of TLIF using the modified distractor handle.

## Figures and Tables

**Table 1:** Demographic and clinical data of LDD patients (*n* = 45) scheduled for TLIF using the modified distractor handle.

Age, year, mean (range)	53 (40–73)
Sex, M/F	30/15
Concomitant lumbar conditions, *n* (%)	
Spinal stenosis with instability	25 (55.6)
Involving single segment	19 (42.2)
Involving two segments	6 (13.3)
Single-segment spondylolisthesis	20 (44.4)
Grade 1	11 (24.4)
Grade 2	9 (20.10)
Fused levels, *n* (%)	
L3-L4	7 (15.6)
L4-L5	23 (51.1)
L5–S1	25 (55.6)
Operative time, min, mean (range)	92.3 ± 34.5 (80–145)
Volume of intraoperative bleeding, min, mean (range)	120.4 ± 40.4 (95–160)
Time length of postoperative hospitalization, *d*, mean (range)	7.3 (5–12)
Duration of follow-up period, mo, mean (range)	17.6 (12–24)

LDD: lumbar degenerative disease; TLIF: transforaminal lumbar interbody fusion.
